# Slow unsteady gait in a population-based cohort: links to ventriculomegaly and INPH-related imaging markers

**DOI:** 10.1186/s12987-026-00830-5

**Published:** 2026-06-12

**Authors:** Jenny Lander, William Hansson, Sanna A. Eklund, Sara Qvarlander, Anders Wåhlin, Bo Traberg Kristensen, Lars-Owe D. Koskinen, Anders Eklund, Jan Malm

**Affiliations:** 1 Department of Clinical Sciences, Neurosciences, Umeå University, Umeå, 901 87 Sweden; 2 Department of Diagnostics and Intervention, Biomedical Engineering and Radiation Physics, Umeå University, Umeå, 901 87 Sweden; 3 Department of Applied Physics and Electronics, Umeå University, Umeå, 901 87 Sweden; 4 Umeå Center for Functional Brain Imaging (UFBI), Umeå University, Umeå, 901 87 Sweden; 5 Department of Neurology, Aalborg University Hospital, Aalborg, 9000 Denmark

**Keywords:** Hydrocephalus, Normal pressure, Magnetic resonance imaging, Gait disorders, Neurologic, Imaging, Three-dimensional, Biomarker

## Abstract

**Background:**

Although clinical evaluation, radiological findings, and supplementary tests are routinely used to diagnose idiopathic normal pressure hydrocephalus (INPH), the large variability in surgical outcomes raises the question of whether diagnostic radiological markers truly reflect the typical clinical gait impairment. Gait disorder (GD) and ventriculomegaly (VM) are both common in older adults and may coincide by chance, potentially contributing to this inconsistency in results. This study aimed to determine the prevalence of typical INPH-related higher-level gait disorder (HLGD), diagnosed independently of imaging, and assess its association with VM and other MRI features.

**Methods:**

In this case-control study, 6467 individuals age 65–84 years were screened for GD per questionnaire. Physicians with experience of neurological GD identified cases with HLGD and matched controls without GD through clinical evaluation of 1047 of these individuals. Subsequently, brain MRI (*n* = 909) or CT (*n* = 98) was performed. After exclusions, 81 with HLGD and 192 controls remained. Radiological hydrocephalus features were compared between the groups.

**Results:**

The prevalence of HLGD in the general older population was 5.8%. For HLGD combined with Evans Index (EI)>0.3, it was 3.7%, and 1.7% had HLGD with Disproportionately Enlarged Subarachnoid space Hydrocephalus (DESH). The estimated prevalence of asymptomatic VM, i.e. EI > 0.3 without GD, was 24%, and 4.1% had asymptomatic VM with DESH. Individuals with HLGD were older and performed worse on cognitive tests. Radiology revealed higher ventricular volumes and EI, and a more acute callosal angle in HLGD versus controls. DESH was also more frequent in HLGD (29% vs 7%, *p* < 0.001). Despite group-level differences, individual overlap was substantial. MRI markers showed poor to moderate ability to discriminate HLGD (AUC 0.614–0.765) from normal gait.

**Conclusions:**

In this population-based case-control study, both HLGD and EI > 0.3 were common. EI > 0.3 occurred more frequently with HLGD, but it was also common in asymptomatic individuals, indicating that ventriculomegaly is not specific to HLGD. Most MRI biomarkers showed limited ability to distinguish HLGD from normal gait, although lateral ventricular volume divided by total intracranial volume (relative ventricular volume) was the most informative. The frequent occurrence of EI > 0.3 and DESH in asymptomatic individuals highlights the need for longitudinal studies to clarify prognosis in these individuals.

**Supplementary Information:**

The online version contains supplementary material available at 10.1186/s12987-026-00830-5.

## Background

The PENS study, a recent randomized controlled trial (RCT) for INPH, confirmed the efficacy of cerebrospinal fluid (CSF) shunting in alleviating symptoms [1], thereby reinforcing the clinical relevance of selecting appropriate candidates for surgery. While patient diagnosis and selection for surgery have always been a central challenge in the management of INPH [2], the availability of high-quality RCT evidence now places renewed emphasis on accurately identifying patients with INPH.

The first challenge is to determine which patients to select for investigation of suspected INPH. The core diagnostic criteria include ventriculomegaly (VM) in combination with a symmetrical, cautious gait [3, 4], often referred to as a higher-level gait disorder (HLGD) [5]. These two features are considered mandatory and are frequently investigated as potential biomarkers for predicting CSF shunt responsiveness [6–11].

Now that the symptom HLGD has been shown to be reversible in a group of patients with INPH [1], the question arises whether this treatment option should be limited to patients with Evans Index (EI)>0.3. Ever since Hakim initially described shunt responsive hydrocephalus, EI > 0.3 has been a core criterion for the diagnosis. It is an appealing and logical assumption, based on the idea that CSF is drained from the dilated ventricles. However, no radiological biomarker has proven reliably predictive for shunt responsiveness [12]. Evidence demonstrates minimal correlation between clinical improvement and either preoperative ventricular size [6, 7] or postoperative changes in ventricular morphology [8, 13, 14]. Recently published data even suggest that among INPH patients with VM, those with a lower EI have a greater chance of improvement [15]. Thus, it is reasonable to ponder whether EI > 0.3 should remain a strict threshold for initiating shunt evaluation, particularly in light of its nearly 90 year old origin. Despite this, it has been suggested we should make surgical decisions based solely on a diagnosis of “possible” INPH, i.e., clinical symptom presentation and EI > 0.3, without relying on additional predictive tests [4, 16]. Such an approach would require that this clinical presentation is highly specific for INPH.

Furthermore, both HLGD and VM have diverse etiologies and are common in older adults [5, 17]. It is therefore reasonable to question whether their co-occurrence could be attributable to chance rather than INPH. Surprisingly little is known about the prevalence of combined VM and HLGD in the general population, how often each occurs in isolation and if there is a true association between the two—representing an important gap in current knowledge.

Given that HLGD and VM are each common in the general population, we hypothesized that their co-occurrence may arise by chance rather than consistently reflect underlying INPH. This large prospective population-based case-control study aimed to determine prevalence of HLGD and VM, defined as EI > 0.3, including their co-occurrence, and to examine whether HLGD is truly associated with quantitative MRI markers of INPH.

## Methods

In summary, in this prospective population-based study, one-third of the older population in Umeå Municipality was randomly selected to complete a questionnaire regarding gait and balance impairments. Individuals who reported gait difficulties, along with sex- and age-matched controls, were invited for clinical examination, which included video recordings and brain MRI. The primary aim was to classify the gait disorder without prior knowledge of subsequent MRI findings, thereby ensuring that the gait and HLGD classification remained independent of imaging results.

### Study population and clinical assessment

**Fig. 1:**
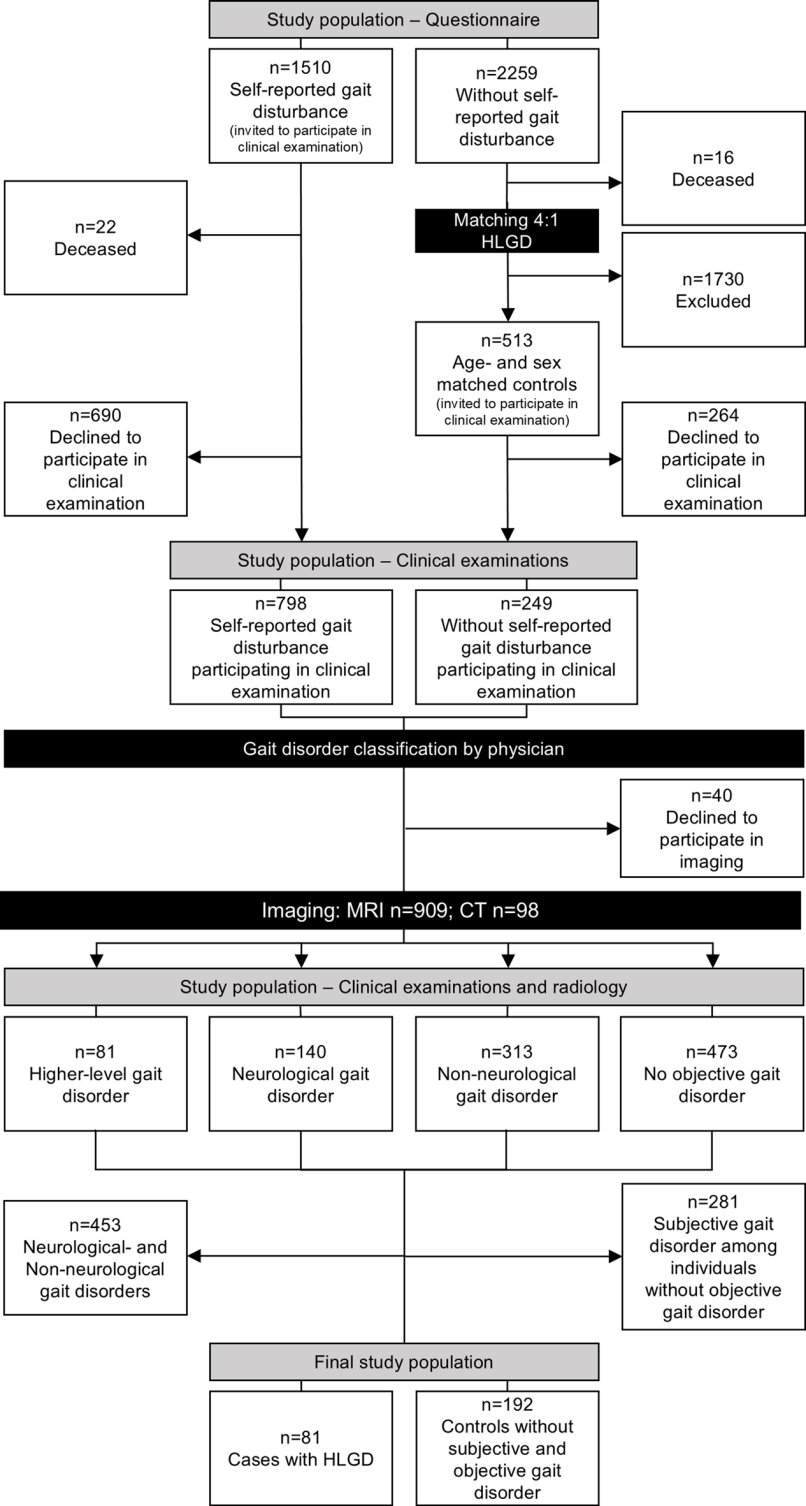
Case- and control flow. Number of survey respondents, individuals with and without subjective and objective gait impairment, individuals with HLGD and controls with normal gait, brain imaging investigations and reasons for exclusions

The study population and inclusion process have been previously described in detail [18]. The case and control selection is illustrated in Fig. 1. Using the Swedish population registry, 6,412 of 18,777 individuals 65–84 years living in Umeå Municipality were randomly invited to participate [19, 20]. The survey was completed by 3,769 individuals (59%), of whom 1510 reported subjective gait impairments and were invited for clinical examination and brain imaging. Among those, 798 accepted the invitation and were assessed by a physician. Standardized interviews covering medical history and a comprehensive neurological examination was conducted by a physician, focusing on gait, nervous system dysfunction and musculoskeletal disorders. Based on the results, i.e. clinical signs of musculoskeletal or neurological GD and/or a known history of such problems, a preliminary GD classification was made by the physician. The gait was also video recorded. Cognition was assessed using the short version of the Montreal Cognitive Assessment (MoCA 5-min protocol) [21].

Later, two physicians independently reviewed the data and video recordings. Preliminary GD classifications were discussed and confirmed or revised by consensus. In cases with multiple contributing factors, these were ranked by relative impact, with the most significant cause used for classification in this study. Diagnoses were established without access to brain MRI or CT. The final clinical diagnoses were: HLGD, *n* = 87; Other neurological gait disorder, *n* = 145; and Non-neurological gait disorder, *n* = 329.

For each participant identified with HLGD, multiple age- and sex matched individuals with a subjective experience of normal gait pattern and speed, according to their questionnaire responses, were invited to undergo an identical clinical examination (*n* = 513); 249 accepted the invitation. Of these, 50 had an objective gait disorder established through clinical examination. In the remaining 199 participants, the physician confirmed normal gait.

After clinical examination and diagnosis, participants were referred for brain MRI (*n* = 909); those unable to undergo MRI received a standard non-contrast CT (*n* = 98). However, six individuals with HLGD and seven with subjective and objective normal gait declined neuroimaging and were therefore excluded. Thus, the final study population comprised 81 individuals with HLGD (73 with MRI and 8 with CT) and 192 controls (184 with MRI and 8 with CT).

### Higher level gait disorder (HLGD)

The symptom HLGD was defined as a symmetrical unsteady gait disorder. In most cases, it was a diagnosis of exclusion, where neither the clinical examination, patient history, nor a review of previous medical records provided another explanation for the symptoms.

The HLGD gait pattern was typically slow, symmetrical and broad-based, characterized by poor foot clearance, short steps, and instability (Table 3). Tasks such as tandem walking and maintaining standing balance were often impaired. Despite these gait abnormalities, muscle strength, motor function, reflexes, sensation, and cranial nerve function were preserved [5, 22]. These characteristics are also in accordance with guideline descriptions of the INPH gait disorder [3, 4].

### MR Imaging

High-resolution 3D MRI T1-weighted images were obtained using a 3T scanner with a 32-channel head coil (Discovery MR 750; GE Healthcare, Milwaukee, Wisconsin). A 3D fast spoiled gradient-echo sequence (BRAVO) was used with a 10° flip angle, 180 slices, 1 mm slice thickness, 256 × 256 mm field of view, 1 mm in-plane resolution, and phase acceleration 2.

### Ventriculomegaly

In this study, VM was analysed according to two definitions used in previous literature, EI > 0.3 [3, 4] and total lateral ventricular volume (VV) >77 ml [17].

### Other neuroimaging features

Calculation of linear and volumetric measurements are described in detail in “Supplementary material 2”. Volumetric measurements were based on automatic segmentation generated with the FreeSurfer image analysis suite version 6.0.0 [23, 24]. Linear measurements were aligned with the bi-commissural line.

The following were estimated in study participants with MRI (HLGD *n* = 73; Controls *n* = 184): EI, Callosal angle (CA), z-EI, the brain-ventricle ratio measured at the level of the anterior commissure (BVR), a modified version of the BVR (mBVR) [23, 24], disproportionately enlarged subarachnoid space hydrocephalus (DESH) [25], DESH score [26], and volumetric measurements of VV and Relative VV (RVV; i.e. VV per total intracranial volume in percent).

Only EI and CA were measured in the individuals who underwent CT (HLGD *n* = 8; Controls *n* = 8).

### Statistical analysis

IBM® SPSS® Statistics 28 (IBM Corp. 2021) and MATLAB (R2021a, Natick, Massachusetts) were used. The significance level was set to *p<*0.05.

Two-sided unpaired t-tests and chi-squared tests were used when appropriate. Receiver operating characteristic curves were determined for the MRI measurements and areas under the curves (AUC) were compared with the z-test. To explore the effect of combining MRI biomarkers, AUC was calculated based on predicted probability determined through a conditional forward logistic regression model including age, sex and the following MRI biomarkers: EI, CA, z-EI, BVR, mBVR, DESH and RVV.

The estimated prevalence of HLGD combined with EI > 0.3 and HLGD combined with DESH (which per definition requires EI > 0.3) in the general older population was calculated through multiplication of the estimated HLGD prevalence (5.8%) in the population (which has been previously described in detail [18]) with the prevalence of EI > 0.3 (64%) and DESH (29%) in the HLGD group:

5.8%*prevalence EI > 0.3 among HLGD (HLGD with EI > 0.3):

$$0.058*0.64 = 0.0371 = 3.7\% $$

5.8%* prevalence DESH among HLGD (HLGD with DESH):

$$0.058*0.29 = 0.0168 = 1.7\% $$

The same approach was used for calculation of EI > 0.3 and DESH in individuals without GD, i.e. asymptomatic VM and asymptomatic VM with features of INPH (AVIM) [27].

The 95% Confidence Intervals were calculated based on the estimated effective sample sizes, i.e. (the identified number of cases within the group)/(estimated prevalence as per the calculations above) = estimated effective sample size [18].

## Results

### Prevalence of HLGD, VM, DESH and the combined condition (i.e., INPH)

The prevalence of individuals above and below commonly used INPH biomarker cut‑offs [4, 17, 28] differed between HLGD and controls (Table 1, chi-squared tests). Biomarkers indicating VM—such as EI > 0.3 and ventricular volume > 77 ml were more common in HLGD than in controls, as were CA < 90 degrees and presence of DESH (which per definition includes EI > 0.3).

**Table 1:** Comparisons of HLGD cases and age- and sex-matched controls relative to MRI biomarker cutoffs for ventricular size

	HLGD, n (%)	Controls, n (%)	OR (95% CI)	p-value*
EI > 0.3^†‡^	52/81 (64)	74/192 (39)	2.86 (1.67—4.90)	<0.001
CA (°) <90^‡^	24/81 (30)	15/192 (8)	4.97 (2.44—10.11)	<0.001
VV (ml) >77^§^	28/73 (38)	12/184 (7)	8.92 (4.21—18.91)	<0.001
z-EI > 0.42^‡^	9/73 (12)	3/184 (2)	8.48 (2.23—32.32)	<0.001
BVR < 1.0^‡^	25/73 (34)	16/184 (9)	5.47 (2.70—11.07)	<0.001
DESH	21/73 (29)	12/184 (7)	5.79 (2.67—12.55)	<0.001

§,Cut-off >77 ml^8^

†,Cut-off based on American/European INPH guidelines^3^

‡,Cut-off based on Japanese INPH guidelines^4^

*p*-value for chi-square test

Abbreviations: VV = total lateral ventricular volume; EI = Evans index; CA = callosal angle; z-EI = z-Evans Index, BVR = brain-ventricle ratio; DESH = Disproportionately Enlarged Subarachnoid space Hydrocephalus; CI = confidence interval; HLGD = Higher-level gait disorder; INPH = Idiopathic Normal Pressure Hydrocephalus; n = number of participants; OR = odds ratio

The prevalence of HLGD in the general older population was 5.8% (95% CI 4.6—7.0%). Frequencies of EI > 0.3 and DESH in HLGD and controls are presented in Table 1. HLGD combined with EI > 0.3 was present in 3.7% (95% CI 2.7—4.7%), corresponding to a frequency of 64% (*n* = 52/81) EI > 0.3 among HLGD. HLGD combined with DESH (which includes EI > 0.3) was present in 1.7% (95% CI 1.0—2.4%) of the general population, corresponding to a frequency of 29% (*n=*21/73) DESH among HLGD.

The prevalence of asymptomatic EI > 0.3 in the general population was 24% (95% CI 19—29%), corresponding to a frequency of 39% (*n* = 74/192) EI > 0.3 among controls. AVIM was present in 4.1% (95% CI 1.8—6.4%) of the population, corresponding to a frequency of 7% (*n=*12/184) DESH among controls.

Hence, risk ratio (RR) for EI > 0.3 in HLGD vs. controls was 1.67 (95% CI 1.31—2.12, *p* < 0.001) and RR for DESH in HLGD vs. controls was 4.4 (95% CI 2.29—8.50, *p* < 0.001).

**Fig. 2:**
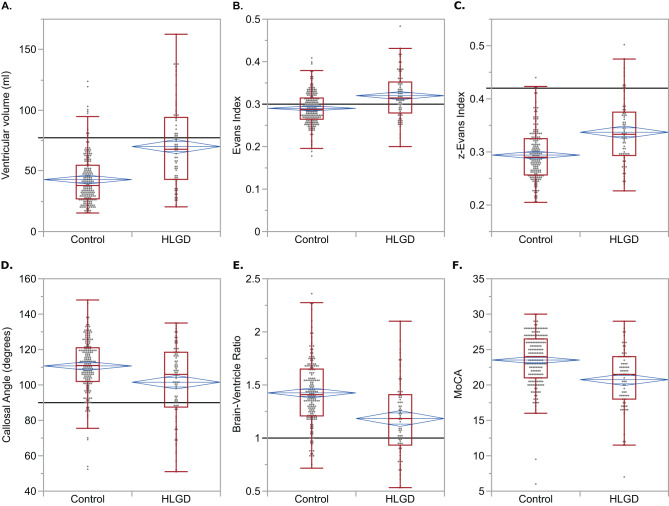
Boxplots of radiological biomarkers for idiopathic normal pressure hydrocephalus diagnostics and 5-minute Montreal cognitive Assessment scores compared between individuals with higher-level gait disorders and gait healthy controls. The red boxes represents interquartile range with median marked by a center line. Whiskers indicate spread of remaining data and blue diamonds show mean values and 95% confidence intervals. Black horizontal lines in panels A-E visualize commonly used cut-offs in INPH diagnostics. Panel **A**: ventricular volume (ml); panel **B**: Evans Index; panel **C**: z-Evans Index; panel **D**: callosal angle (degrees); panel **E**: brain-ventricle ratio: panel **F**: 5-minute Montreal Cognitive Assessment (MoCA)

Based on ventricular volume, 38% (*n* = 28/73) of HLGD cases had enlarged ventricles (i.e. >77 ml), versus only 7% (*n* = 12/184) of controls (Table 1). The CA and BVR showed similar patterns (Fig. 2D–E and Table 1).

Men had higher EI than women (two-sided t-test, mean±SD, men vs. women: 0.307 ± 0.044, *n* = 165 vs. 0.288 ± 0.044, *n* = 108; *p* = 0.001). In comparisons of VV and RVV the difference between sexes remained (two-sided t-test, mean±SD, VV men vs. VV women: 56 ± 29 ml vs. 43 ± 23 ml; *p* < 0.001; RVV men vs. RVV women: 3.2 ± 1.5 vs. 2.8 ± 1.4; *p* = 0.024). However, when the difference between sexes in EI, VV and RVV was explored within different age groups, a discrepancy was seen with larger difference within the age group over 79 years (supplementary Figure 1) and no difference in the youngest age group (EI, VV and RVV) or in RVV in ages 75–79 years. Individuals with EI > 0.3, among HLGD and controls, were older than those without EI > 0.3 (two-sided t-test, mean±SD, individuals with EI > 0.3 vs. EI ≤ 0.3: 76.6 ± 4.50 years, *n* = 116 vs. 74.6 ± 4.12 years, *n* = 141; *p* < 0.001).

### INPH imaging features and their predictive power of HLGD

Mean values for the ventricular measures are presented in Fig. 2 and Table 2. Although group differences were observed across all neuroimaging variables, Fig. 2 highlights considerable overlaps between HLGD and controls. Individuals with HLGD showed larger mean values for VV, EI, z-EI and DESH score (which includes EI > 0.3), and smaller mean values for CA, BVR and mBVR, compared with controls. These differences remained significant after adjustment for age, sex, cognition, and total brain volume. While mean values reveal larger ventricles in the HLGD group, several individuals in both groups exhibited similarly enlarged ventricles (Fig. 2a–c). In an analysis of DESH score where the EI > 0.3 variable was excluded from the total score, the difference between HLGD and controls remained (two-sided t-test, mean±SD, HLGD vs. controls: 2.8 ± 2.4, *n* = 73 vs. 1.2 ± 1.6, *n* = 184; *p* < 0.001). I.e. individuals with HLGD had more typical radiological signs of hydrocephalus, not merely EI > 0.3 associated with general brain atrophy, than gait-healthy controls.

**Table 2:** Mean values and differences in MRI biomarkers between HLGD cases and age- and sex-matched controls, including area under the receiver operating characteristic curve (AUC) values as predictors of HLGD

**Variable**	**HLGD, mean±SD (n)**	**Controls, mean±SD (n)**	**Mean difference (95% CI)**	**p-value**	**AUC** **(95% CI)**	**p-value**
VV, ml	70 ± 34 (73)	43 ± 21 (184)	27 (19—36)	<0.001	0.755 (0.687—0.823)	<0.001
RVV	4.1 ± 1.7 (73)	2.6 ± 1.1 (184)	1.5 (1.1—1.9)	<0.001	0.765 (0.700—0.830)	<0.001
DESH score	3.6 ± 2.8 (73)	1.7 ± 2.0 (184)	1.970 (1.36—2.58)	<0.001	0.723 (0.652—0.794)	<0.001
EI	0.32 ± 0.05 (81)	0.29 ± 0.04 (192)	0.030 (0.017—0.043)	<0.001	0.670 (0.596—0.744)	<0.001
CA, °	101 ± 22 (81)	111 ± 15 (192)	−9.1 (−14.4—3.8)	<0.001	0.616 (0.538—0.693)	0.003
z-EI	0.34 ± 0.06 (73)	0.29 ± 0.05 (184)	0.043 (0.027—0.059)	<0.001	0.707 (0.634—0.780)	<0.001
BVR	1.18 ± 0.35 (73)	1.43 ± 0.31 (184)	−0.24 (−0.33—0.15)	<0.001	0.697 (0.623—0.771)	<0.001
mBVR	1.32 ± 0.41 (73)	1.63 ± 0.40 (184)	−0.31 (−0.42—0.20)	<0.001	0.708 (0.636—0.780)	<0.001

Abbreviations: HLGD = Higher-level gait disorder; VV = total lateral ventricular volume; RVV = total lateral ventricular volume per total intracranial volume; EI = Evans index; CA = callosal angle; z-EI = z-Evans Index; BVR = brain-ventricle ratio; mBVR = modified version of the brain-ventricle ratio; DESH = Disproportionately Enlarged Subarachnoid space Hydrocephalus; CI = confidence interval; SD = standard deviation; AUC = area under the receiver operating characteristic curve; n = number of participants

**Fig. 3:**
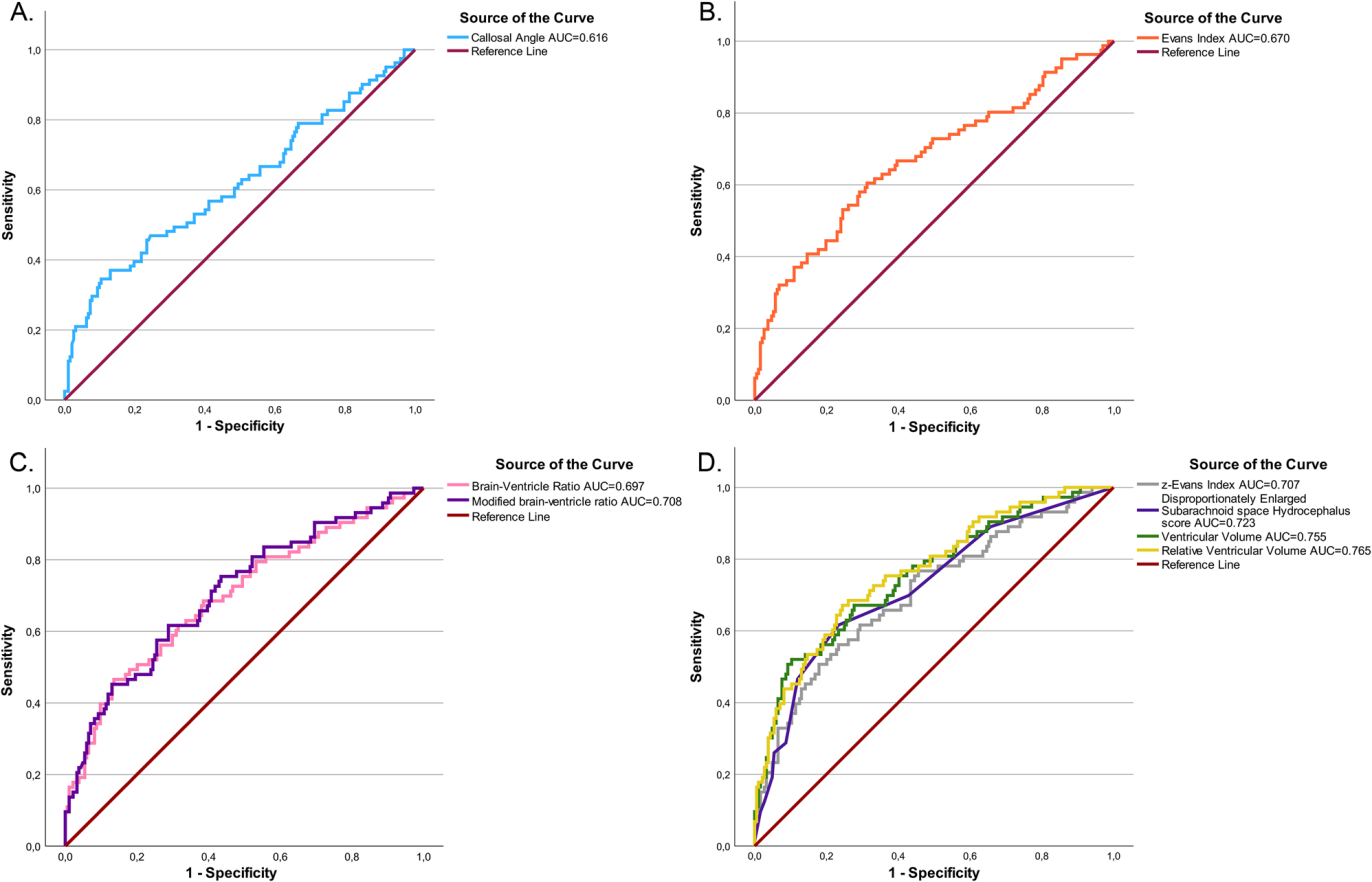
Receiver operating characteristic curves for different radiological biomarkers used for idiopathic normal pressure hydrocephalus diagnostics, illustrating their predictive power for higher-level gait disorders (HLGD). Panel **A** and **B** show curves calculated based on CT and MRI data (*n* = 273) while curves in panel **C** and **D** are based on MRI data only (*n* = 257). Panel **A** and **C** show curves for data where lower numbers represent higher likelihood for HLGD. Panel **B** and **D** show curves for data where higher numbers represent higher likelihood for HLGD

As shown in Fig. 3 and Table 2, ventricular size measured with volume segmentations and linear measurements could predict HLGD with poor to acceptable discrimination (area under receiver operating curve (AUC) range: 0.614—0.765). In a comparison of the AUC values, VV was a better predictor for HLGD than all linear measurements (*p* < 0.050). There was no difference in AUC between the different linear measurements. The cut-off value providing the best Youden Index for VV (0.417) was 67 ml with a sensitivity of 52% and a specificity of 90%. In a conditional logistic regression model of the linear measurements, DESH and RVV, only EI and RVV remained statistically significant, providing additional information to the model. These two measurements combined gave an AUC of 0.790 (95% CI 0.724—0.847, *p* < 0.001), with a best combined sensitivity of 71% and specificity of 72%.

### Clinical features of HLGD

Individuals with HLGD performed worse on the 5-min MoCA test compared with controls (Fig. 2f; two-sided t-test, mean±SD: 21 ± 4.2 points, *n* = 81 vs 24 ± 3.6 points, *n* = 192; *p* < 0.001). As shown in Table 3, individuals with HLGD exhibited slow gait speed, a symmetrical broad-based gait with shuffling steps, and often impaired balance. There was no difference in frequency of males and females between HLGD and controls (chi-square test women with HLGD vs controls: 38% (*n* = 31/81) vs. 40% (*n* = 77/192); *p* = 0.777). Individuals with HLGD had slightly higher mean age compared with controls (two-sided t-test, mean±SD: 77 ± 4.8 years, *n* = 81 vs. 75 ± 3.2 years, *n* = 192; *p* < 0.001).

**Table 3:** Features of gait in higher-level gait disorders

Gait feature	HLGD, n (%)	Controls, n (%)	p-value
Slow gait	71/81 (88)	1/192 (0.5)	<0.001
Symmetrical gait	54/81 (67)	191/192 (99.5)	<0.001
Broad based gait	51/81 (63)	0/192 (0.0)	<0.001
Shuffling gait	37/81 (46)	0/192 (0.0)	<0.001
Unsteady narrow stance balance, eyes closed	26/81 (32)	13/191 (6.8)	<0.001
Oppositional paratonia	24/77 (31)	32/189 (17)	0.010
Limited passive hip joint movement	23/77 (30)	29/191 (15)	0.006

Abbreviations: *n* = number of participants

## Discussion

This study aimed to determine the prevalence of typical INPH gait disorder (HLGD), diagnosed independently of imaging findings, and to examine its association with VM and other INPH-related imaging features. Both HLGD and VM were found to be highly prevalent in the general older population, with VM being more common in individuals with HLGD than in those without gait impairment. However, substantial overlap existed, as VM was frequently observed in both groups, likely complicating INPH diagnostics and prognostication prior to shunt surgery. Hence, MRI markers showed limited ability to discriminate HLGD from controls. These results are of great importance since no previous study has evaluated the ability of these radiological biomarkers to discriminate the INPH gait disorder, i.e. HLGD, from normal gait. Since a random selection of individuals from the population were invited to participate, and exclusion criteria were few, the results are also highly generalizable to the older population in Sweden.

### Idiopathic normal pressure hydrocephalus

Nearly six decades after Hakim and Adams first described INPH, the PENS study (recently published RCT) confirmed that shunt treatment in INPH significantly improves gait speed [1]. In this context, the analysis in our study focuses specifically on the relationship between the typical INPH gait disorder, i.e. HLGD, and VM, in a population-based cohort. Importantly, our study does not evaluate the full INPH symptom-triad, nor does it assess shunt responsiveness. Therefore, the findings should not be interpreted as a direct test of INPH diagnostic pathways or surgical selection criteria. However, our findings highlight why INPH has been difficult to define as a homogeneous disorder: EI > 0.3 did not reliably indicate HLGD, and controls without HLGD could still exhibit EI > 0.3. This illustrates that both HLGD and VM are relatively common findings in the general population and, although associated at the group level, do not consistently co-occur in individual cases.

While current guidelines recommend supplementary tests to identify surgical candidates, the Japanese guidelines also allow surgery based solely on typical clinical and radiological features [3, 4]. The observation that HLGD occurs both with and without VM, and that established MRI biomarkers show limited ability to predict HLGD, suggest that radiological markers alone may not be sufficient to distinguish HLGD from normal gait in a population-based setting. This highlights the need for further RCTs to clarify whether clinical and radiological criteria alone can reliably guide management, or whether supplementary testing remains essential.

According to guideline definitions, the combination of HLGD and EI > 0.3 fulfils the diagnostic criteria for “possible” INPH [3] and it was present in 3.7% of the population while 1.7% had “possible” INPH with support of DESH. Recent INPH prevalence studies, using similar definitions, show similar prevalence rates of “probable” (2.1—3.7%) [29–31] and “possible” (7.3%) [30] INPH. A large discrepancy is seen to the number of actually shunted patients (3.7 per 100,000 and year in Sweden) [32], resulting in suggestions that INPH is underdiagnosed or undertreated [29, 30]. However, this classification, the combination of HLGD and EI > 0.3, does not imply that these individuals have clinical INPH or would be candidates for shunt evaluation. It should be emphasized that “possible” INPH is an operational classification and does not imply surgical eligibility. Symptom severity and comorbidity, both crucial for surgical decision-making, were not assessed in this study or in previous prevalence studies. Future epidemiological studies should therefore extend beyond diagnostic categorization to systematically evaluate surgical candidacy, incorporating patient preference and adherence to current surgical guidelines.

### Asymptomatic ventriculomegaly

The high prevalence of asymptomatic EI > 0.3 and AVIM (asymptomatic VM, i.e. EI > 0.3, combined with DESH [27]) observed in our cohort raises questions regarding the longitudinal development of INPH. Does asymptomatic VM and AVIM represent congenital conditions, or is it a precursor stage that may progress to INPH over time, as implied in previous research [27]? If the latter is true, could symptomatic progression be prevented by early shunt intervention or drugs that have not yet been developed?

The international hydrocephalus guidelines define VM as an EI > 0.3 [3, 4]. In this study, EI > 0.3 was common in the general population, but the prevalence of “possible” INPH, i.e. HLGD combined with VM [3, 4], varied depending on the radiological measurement used. It was frequent when VM was measured with EI, while large ventricular volume was less common (Fig. 2). The previously used cut-off of >77 ml [17] essentially excluded VM in controls. We concluded that the cut-off with best Youden index was 67 ml; it showed moderate sensitivity (49%), indicating that HLGD can originate from other causes than hydrocephalus, but high specificity (91%), suggesting that few individuals with normal gait have larger ventricular volume. One previous study by Constantinescu et al. made an extensive job correlating symptoms with morphological imaging markers, presented in their supplementary material [33]. They found no correlations between imaging markers and gait speed or balance, however, the overall evaluation of whether these symptoms reflected an INPH-resembling gait disorder was not performed. Considering the substantial overlap in EI > 0.3 between HLGD and controls, together with the low AUC values for radiological hydrocephalus measurements for identifying a typical gait disorder, our findings suggest that the currently used combination of symptoms and radiological criteria is not sufficiently specific to uniquely characterize INPH. In clinical practice, overlap between age-related atrophy, white matter disease and mixed neurodegenerative pathology is a frequent challenge. Such overlap may also blur the relationship between VV and HLGD. In this study, several analyses were made to correct for atrophy (corrections for total brain volume, exclusion of EI > 0.3 from the DESH score variable and measures of mBVR and RVV), and the association between the MRI biomarkers and HLGD still remained, suggesting a true association. However, even in a carefully phenotyped cohort, these common pathologies in older adults makes it difficult to determine whether ventricular morphology truly reflects hydrocephalus-related pathology or more ubiquitous degenerative processes. This indicates that additional or more refined diagnostic tools are needed to better distinguish INPH from other conditions with similar clinical and imaging profiles.

EI is simple to measure, whereas volume is more precise but less accessible. Hence, longitudinal studies evaluating symptom progression in asymptomatic VM and population-specific reference values, accounting for sex, age, and ethnicity, are needed. Also, today, automatic brain volumetric analyses are used in research but not usually integrated in clinical routine. To enhance hydrocephalus evaluations such volumetric measurements could be integrated as standard procedure. However, even though they performed better than the classical measurements, both VV and RVV also had moderate predictive power for HLGD.

### The typical INPH gait disorder without ventriculomegaly

Greater awareness among health care professionals of HLGD and related conditions is essential for accurate referrals, efficient diagnostic work-up, and to reduce the socio-economic burden. For instance, in this study, one-fifth of individuals who considered themselves normal walkers were diagnosed with gait disorders, including two with HLGD. The widespread belief that gait decline is a normal part of aging currently leads to dismissal of isolated HLGD.

Since the cut-off of EI > 0.3 is both a diagnostic criterion for INPH and a requirement in preoperative evaluations for shunt surgery [3, 4], clarifying its relationship with the INPH gait disorder is crucial. Studies have shown that there is a weak correlation between ventricular size and clinical improvement after shunt surgery [6–8], but lower EI has in one study been associated with improved outcome [15]. Our study also showed that as many as one third of individuals with HLGD did not fulfil diagnostic criteria for hydrocephalus, i.e. they had EI ≤ 0.3. In many cases, no obvious cause for the symptom was found. Studies on shunt surgery for individuals with HLGD and EI just below 0.3 are lacking. Considering the high prevalence of HLGD of unknown cause without EI > 0.3 in this study, our findings suggest that the clinical relevance of the current cutoff warrants further evaluation. On the other hand, other causes of HLGD exist, such as microvascular disease and, in some cases, parkinsonian disorders [5]. We have previously published data from this population showing that as many as 87% of the individuals with HLGD did not know the cause for their gait disorder, and the physician was also unable to determine the cause based solely on the initial physical examination [18]. These observations underscore the challenge of identifying the underlying cause of HLGD in many individuals and highlight the need for reliable diagnostic tools that can help distinguish INPH from other conditions with similar gait presentations.

### Prevalence and clinical profile of higher-level gait disorders

This study confirms a well-established fact: gait disorders are common among older people [34, 35]. In the present cohort, nearly 40% of individuals exhibited gait disorder, regardless of underlying cause. We found HLGD to be among the most frequent causes, affecting nearly one in seventeen older adults [18]. Yet, despite its high prevalence, it has received remarkably little scientific attention [5]. HLGD is a clinical diagnosis, based on gait and balance assessment, with otherwise often unremarkable neurological findings [5]. We have previously shown that individuals with HLGD have lower quality of life, more depressive symptoms, and reduced gait confidence compared with those without gait disorders [18]. HLGD was also characterized by poorer performance on cognitive testing compared with controls, in similarity with previous INPH literature [5]. These findings help to further define the clinical profile of HLGD, and may also provide insights relevant to associated conditions, such as INPH.

### Strengths and limitations

This study was strengthened by its prospective design, investigating a substantial and representative sample from the general population, and ensuring that the gait disorder was determined unbiased of imaging findings. Exclusion criteria were few, and clinical examinations were performed by physicians with experience in the field. On the other hand, clinical gait evaluations are subjective, and physicians may interpret gait characteristics differently. To minimize this variability, we used video recordings reviewed by two physicians who reached decisions by consensus. Also, in this study, the classical INPH symptom triad including cognitive decline and urinary incontinence was not regarded, only the gait symptom. However, while cognitive decline and urinary incontinence in combination with EI > 0.3 are enough to receive a diagnosis of “possible” INPH [3, 4], the recently performed RCT on shunting for INPH did not show improvement in either of these symptoms following shunt surgery [1]. Although more expensive and time-consuming, MRI was used instead of CT in this study, which is common in previous studies. MRI, including volumetric analysis, provides superior diagnostic information and is increasingly used in the evaluation of patients with suspected hydrocephalus.

## Conclusions

In conclusion, both HLGD and VM are common in the older population and associated at the group level. However, the large overlap in radiological findings between individuals with and without HLGD limits the ability of established INPH MRI biomarkers to distinguish HLGD from normal gait in this population-based cohort. This challenges current assumptions, and suggest that additional phenotyping and improved diagnostic tools are needed.

## Electronic supplementary material

Below is the link to the electronic supplementary material.

Supplementary material 1

Supplementary material 2

## Data Availability

Upon request from a qualified investigator, anonymized raw data can be made available.

## Abbreviations

AVIM: Asymptomatic ventriculomegaly with features of INPH
AUC: Area under the receiver operating characteristic curve
BVR: Brain-Ventricle Ratio
CA: Callosal Angle
CSF: Cerebrospinal Fluid
CT: Computed Tomography
DESH: Disproportionately Enlarged Subarachnoid space Hydrocephalus
EI: Evan Index
HLGD: Higher-Level Gait Disorder
INPH: Idiopathic Normal Pressure Hydrocephalus
MoCA: Montreal Cognitive Assessment
MRI: Magnetic Resonance Imaging
VM: Ventriculomegaly
VV: Total lateral Ventricular Volume
RVV: Relative Ventricular Volume
