# Supplementary figures and images for: Slow unsteady gait in a population-based cohort: links to ventriculomegaly and INPH-related imaging markers
Source: Fluids Barriers CNS. 2026 Jun 12;23:80. doi: 10.1186/s12987-026-00830-5 (PMC13270810; doi:10.1186/s12987-026-00830-5)

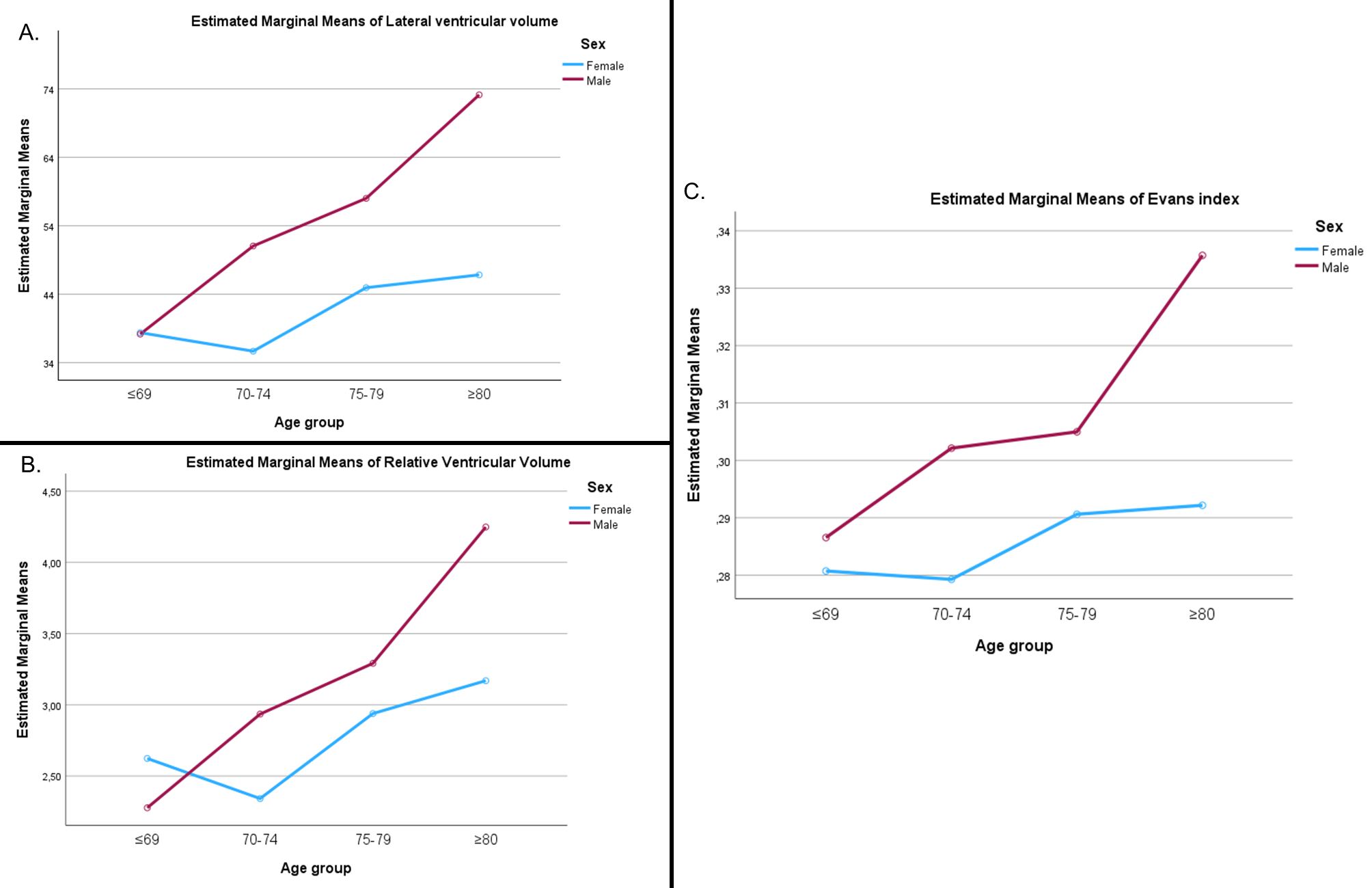

Supplement: Supplementary file 1 — Supplementary material 1 [file 12987_2026_830_MOESM1_ESM.tif]
