# Supplementary material for: Slow unsteady gait in a population-based cohort: links to ventriculomegaly and INPH-related imaging markers
Source: Fluids Barriers CNS. 2026 Jun 12;23:80. doi: 10.1186/s12987-026-00830-5 (PMC13270810; doi:10.1186/s12987-026-00830-5)
Supplement: Supplementary file 2 — Supplementary material 2 [file 12987_2026_830_MOESM2_ESM.docx]

### Appendix

### Linear measurements

All manually measured variables are referred to as linear measurements and described below. They were all measured in T1-weighted images, aligned with the bi-commissural line.

The *bi-commissural line* was defined in midline sagittal images, passing through the superior edge of the anterior commissure and the inferior edge of the posterior commissure (tangential intercommissural line according to Talaraich-Nowinski landmarks)^1^.

*Evan’s index (EI)^2^* was measured in axial images and defined as the ratio between the maximum width of the frontal horns of the lateral ventricles (FHW) and the parallel maximum biparietal diameter (BPD) in the same plane. The maximum FHW was identified by scanning through the images and visually identifying the plane with the maximum FHW. Three consecutive slices were measured and the largest FHW was used. EI was measured by two evaluators (S.E. and J.L.; *ICC*=0.96, 95% CI: 0.93-0.98, *n*=40).

The *corpus callosum angle (CA)* was measured by one evaluator (S.E.; intra-operator *ICC*=0.98, 95% CI: 0.97-0.99, *n*=59) in coronal images at the level of the posterior commissure and defined as the angle of the corpus callosum measured in degrees.^3, 4^

The *z-EI* was measured by three evaluators (S.E., J.L. and J.M.; *ICC*=0.98, 95% CI: 0.96-0.99, *n*=27) in coronal images at the level of the anterior commissure and defined as the ratio between the height of the largest frontal horn and the height of the supratentorial intracranial space, from the posterior sellae turcica to the scull.^5, 6^

The *brain-ventricle ratio (BVR)* was measured by three evaluators (S.E., J.L. and J.M.; *ICC*=0.98, 95% CI: 0.97-0.99, *n*=27) in coronal images at the level of the anterior commissure and defined as the ratio between the brain above the lateral ventricle with the largest frontal horn and the height of the largest frontal horn.^5, 7^

A *modified version of the BVR (mBVR)* was measured by three evaluators (S.E., J.L. and J.M.; *ICC*=0.98, 95% CI: 0.97-0.99, *n*=27) in coronal images at the level of the anterior commissure and defined as the ratio between the intracranial space above the lateral ventricle with the largest frontal horn and the height of the largest frontal horn. The mBVR was developed with the aim to minimize the effect from brain atrophy, focusing the differentiating effect on enlargement of the lateral ventricles in the craniocaudal direction.

*Disproportionately Enlarged Subarachnoid space Hydrocephalus* (DESH) was determined as present or absent by two evaluators (J.M. and W.H.; *ICC*=0.86, 95% CI: 0.82-0.89, *n*=244). It was evaluated in coronal images, in the area between the anterior and posterior commissure, in individuals with EI>0.3. It was defined as present if flattened convexity sulci in relation to dilated Sylvian fissures was observed^8^.

*DESH score* was calculated according to the definition by Shinoda et al.^9^ Dilated sylvian fissures, tight high convexity and focal sulcal dilation was evaluated in coronal images, in the area between the anterior and posterior commissure by two evaluators (J.M. and W.H.). EI and CA were measured as previously described and divided into groups as per the DESH scale definition (*ICC J.M. vs W.H.*=0.95, 95% CI: 0.94-0.96, *n*=244).

### Volumetric measurements

Volumetric measurements were based on automatic segmentation generated with the FreeSurfer image analysis suite version 6.0.0.^10, 11^ T1-weighted images, corrected for spatial inhomogeneity in signal intensity, were analyzed. All segmentations (*n*=257) were manually screened for large errors. For improvement, the segmentation was rerun with a “big ventricles” option for 8 of the participants. Persisting large errors, affecting the areas of interest in this study (total brain volume and ventricular volume), were manually corrected (*n*=15). Volumes were calculated as the sum of the volumes of all voxels included in the relevant segmentation labels. Separate labels for the right and left superior and inferior lateral ventricles, choroid plexus, and total brain volume were provided.

The *total lateral ventricular volume* (VV) was calculated as the sum of the inferior and superior lateral ventricular volumes and the choroid plexus volume.

The *Relative ventricular volume* (RVV) was calculated as VV per estimated total intracranial volume and presented in percent.

**References**

1. Nowinski WL. Modified Talairach landmarks. Acta Neurochir (Wien) 2001;143:1045-1057.

2. Evans WA. An encephalographic ratio for estimating ventricular enlargement and cerebral atrophy. Archives of neurology and psychiatry (Chicago) 1942;47:931-937.

3. LeMay M, New PF. Radiological diagnosis of occult normal-pressure hydrocephalus. Radiology 1970;96:347-358.

4. Ishii K, Kanda T, Harada A, et al. Clinical impact of the callosal angle in the diagnosis of idiopathic normal pressure hydrocephalus. Eur Radiol 2008;18:2678-2683.

5. Nakajima M, Yamada S, Miyajima M, et al. Guidelines for Management of Idiopathic Normal Pressure Hydrocephalus (Third Edition): Endorsed by the Japanese Society of Normal Pressure Hydrocephalus. Neurol Med Chir (Tokyo) 2021;61:63-97.

6. Yamada S, Ishikawa M, Yamamoto K. Optimal Diagnostic Indices for Idiopathic Normal Pressure Hydrocephalus Based on the 3D Quantitative Volumetric Analysis for the Cerebral Ventricle and Subarachnoid Space. AJNR Am J Neuroradiol 2015;36:2262-2269.

7. Yamada S, Ishikawa M, Yamamoto K. Comparison of CSF Distribution between Idiopathic Normal Pressure Hydrocephalus and Alzheimer Disease. AJNR Am J Neuroradiol 2016;37:1249-1255.

8. Hashimoto M, Ishikawa M, Mori E, Kuwana N, Study of Ioni. Diagnosis of idiopathic normal pressure hydrocephalus is supported by MRI-based scheme: a prospective cohort study. Cerebrospinal Fluid Res 2010;7:18.

9. Shinoda N, Hirai O, Hori S, et al. Utility of MRI-based disproportionately enlarged subarachnoid space hydrocephalus scoring for predicting prognosis after surgery for idiopathic normal pressure hydrocephalus: clinical research. J Neurosurg 2017;127:1436-1442.

10. Fischl B, Salat DH, Busa E, et al. Whole brain segmentation: automated labeling of neuroanatomical structures in the human brain. Neuron 2002;33:341-355.

11. FreeSurfer [online]. Available at: <http://surfer.nmr.mgh.harvard.edu/>. Accessed February 3rd.
